# Rare Failures of DNA Bar Codes to Separate Morphologically Distinct Species in a Biodiversity Survey of Iberian Leaf Beetles

**DOI:** 10.1371/journal.pone.0074854

**Published:** 2013-09-05

**Authors:** Andrés Baselga, Carola Gómez-Rodríguez, Francisco Novoa, Alfried P. Vogler

**Affiliations:** 1 Departamento de Zoología, Facultad de Biología, Universidad de Santiago de Compostela, Santiago de Compostela, Spain; 2 Department of Life Sciences, Natural History Museum, London, United Kingdom; 3 Division of Biology, Imperial College London, Ascot, United Kingdom; University of Arkansas, United States of America

## Abstract

During a survey of genetic and species diversity patterns of leaf beetle (Coleoptera: Chrysomelidae) assemblages across the Iberian Peninsula we found a broad congruence between morphologically delimited species and variation in the cytochrome oxidase (*cox1*) gene. However, one species pair each in the genera *Longitarsus* Berthold and *Pachybrachis* Chevrolat was inseparable using molecular methods, whereas diagnostic morphological characters (including male or female genitalia) unequivocally separated the named species. Parsimony haplotype networks and maximum likelihood trees built from *cox1* showed high genetic structure within each species pair, but no correlation with the morphological types and neither with geographic distributions. This contrasted with all analysed congeneric species, which were recovered as monophyletic. A limited number of specimens were sequenced for the nuclear 18S rRNA gene, which showed no or very limited variation within the species pair and no separation of morphological types. These results suggest that processes of lineage sorting for either group are lagging behind the clear morphological and presumably reproductive separation. In the Iberian chrysomelids, incongruence between DNA-based and morphological delimitations is a rare exception, but the discovery of these species pairs may be useful as an evolutionary model for studying the process of speciation in this ecological and geographical setting. In addition, the study of biodiversity patterns based on DNA requires an evolutionary understanding of these incongruences and their potential causes.

## Introduction

DNA sequence data have been successfully used to describe patterns of biodiversity and assemblage variation in space and time [1–3]. In particular, short mitochondrial DNA fragments, such as the Cytochrome Oxidase Subunit I (*cox1*) ‘barcode’ marker [4], have been used to perform extensive sequencing of full communities allowing the description of diversity patterns at species and genetic levels [5]. DNA bar coding follows from earlier studies showing that sequence variation in the *cox1* gene is broadly concordant with existing taxonomic estimates and biogeographical distributions in poorly known groups [6,7]. Sequence-based community analysis therefore has the potential to speed up the systematic assessment of biodiversity patterns, even in ecological systems for which taxonomic information is very limited. This approach also provides the possibility to test statistical patterns of diversity based on the genetic variation of mtDNA haplotypes and therefore has been termed haplotype-based macroecology [3].

The usefulness of mitochondrial community analyses for describing broad biodiversity patterns is dependent on a close match between morphologically and genetically delimited species. However, literature surveys have shown that >20% of species pairs exhibit some level of incongruence [8]. One of the main causes for incongruence is that gene flow generally affects mtDNA to a greater extent than nuclear markers [9,10], which is frequently unexplained, but may be driven by adaptive introgression of mtDNA [11], sex-biased asymmetries [12] or germ-line infecting pathogens that distort the inheritance of mtDNA [13]. In addition, poor lineage separation due to short divergence times may result in lack of diagnosability and non-monophyly of established species, although this effect should be reduced in mtDNA compared to nuclear markers because of increased drift effects from smaller effective population size of mtDNA [8]. Geographic differentiation may also confound the recognition of species boundaries if intra-specific variation is high due to comparatively ancient biogeographic subdivision relative to speciation events [14,15]. This latter phenomenon in particular affects the performance of methods for species recognition based on sequence divergence, since the difference between the amount of intra- and interspecific divergences (i.e. the ‘DNA barcoding gap’) is reduced when working at large geographic scales [16].

For the practice of mtDNA-based biodiversity surveys the existence of such discrepancies leaves the question about the degree to which a single marker would mislead these studies. Biodiversity patterns are the summation of many species distributions, and haplotype-derived patterns might be confounded if mtDNA groups do not reflect true species limits or the species’ geographic extent. For example, in the leaf beetle *Timarchagoettingensis* complex in the Iberian Peninsula both mitochondrial and nuclear markers showed two deeply subdivided lineages but their geographic ranges differed widely [17], which potentially misleads the analysis of geographical diversity patterns. Similarly, mtDNA variation may be insufficient to recognize species if lineages are not subdivided or if subdivision is very shallow. If these various forms of discrepancy with morphological species circumscription were common, it would preclude the proper recognition of biodiversity patterns, including the analysis of decreasing similarity in communities with geographic distance [5]. Therefore, knowing the frequency of incongruences in mtDNA vs. nuclear DNA or morphology is necessary to provide robustness to the haplotype-based macroecology.

During a study of genetic variation of leaf beetles (Coleoptera, Chrysomelidae) assemblages in the Iberian Peninsula we found a general broad overlap between morphologically delimited species recognized by current taxonomy [18–20] and putative species-level groupings estimated from mitochondrial sequences. This broad congruence, involving >200 morphologically-based species, will allow us to assess patterns of species and genetic turnover across the Iberian communities in an integrated framework [5]. However, we also encountered several cases of incongruence in morphological and mtDNA-based species limits. In most of them, morphological species were further split in two or more units by molecular methods, in line with previous estimates suggesting that undescribed leaf beetle species are probably to be discovered in Southern European countries [21]. More remarkably, we observed two pairs of morphologically distinct species that were recovered by mtDNA as a single unit, namely *Pachibrachys* *azureus* Suffrian 1848 and *P* *. regius* Schaufuss 1862, and *Longitarsusatricillus* Linnaeus 1761 and *L* *. bedelii* Uhagon 1887. The purpose of this paper is to characterize these cases of incongruence of morphology and mtDNA, and to discriminate among alternative evolutionary explanations.

## Material and Methods

### Sampling and morphological taxonomy

**Figure 1:**
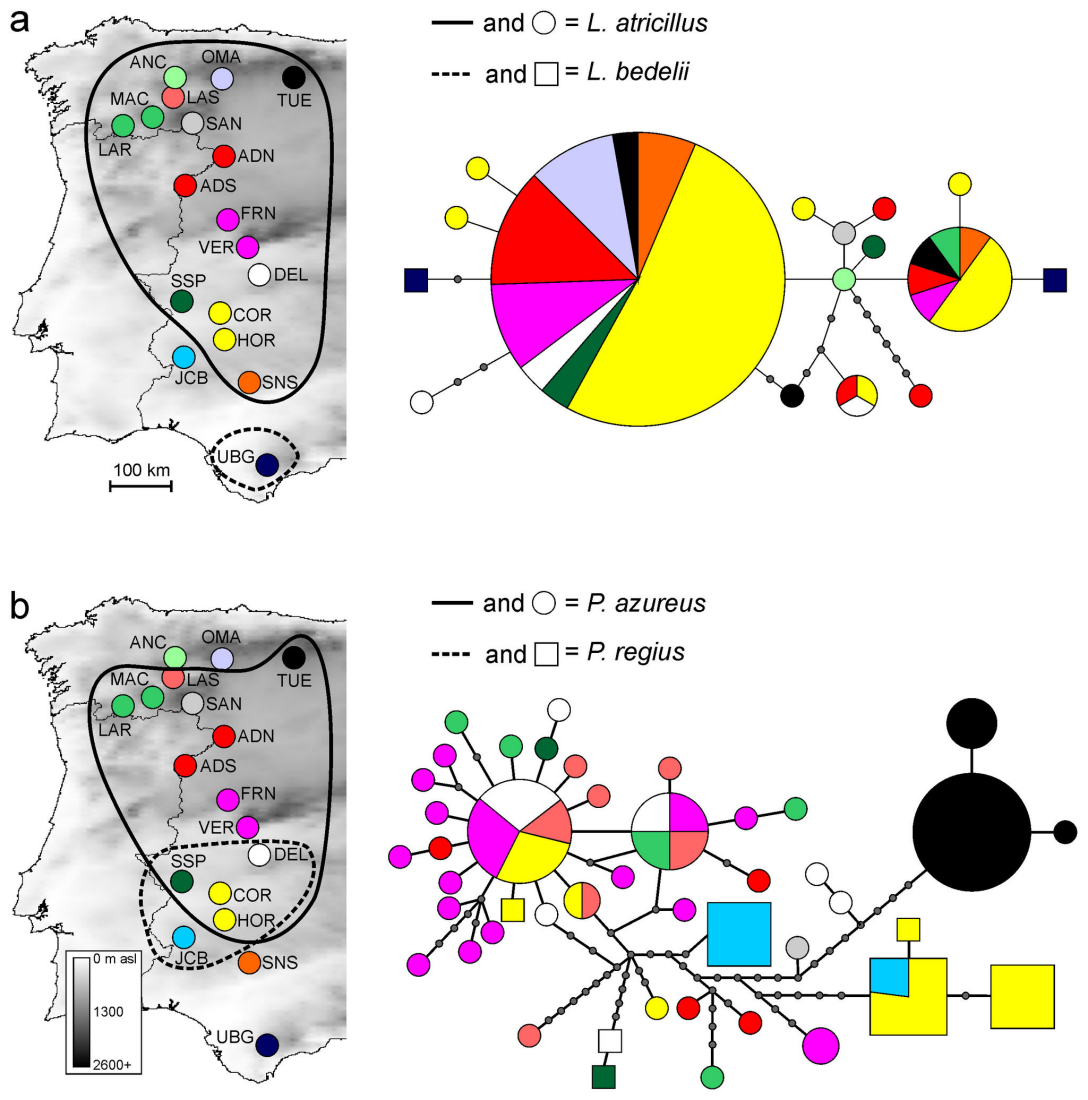
Geographical distribution of sampled localities and haplotype networks. a) Haplotype network for *L* *. atricillus* and *L* *. bedelii* . b) Haplotype network for *P* *. azureus* and *P* *. regius* . Colours in the network correspond to the localities in the map, and the size of the nodes corresponds to number of individuals. Note that distribution ranges reflect our collections, not real distributions.

Specimens of *L* *. atricillus* , *L* *. bedelii* , *P* *. azureus* or *P* *. regius* were collected in 18 localities in Spain (Tables 1 and 2) in April-June 2010. Two additional localities visited in the course of the community study did not yield these species. These localities covered the full South–North gradient in the Iberian Peninsula and were separated from the closest locality by a minimum of 34.8 km (ANC-LAS) and a maximum of 149.5 km (UBG-SNS) (Figure 1). Sampling localities spanned an altitudinal range between 250 and 1270 m above see level. Each locality was intensively sampled, by sweeping and beating all types of vegetation, including trees, shrubs and herbs, for 20 sampling periods of 30 minutes (18 sampling units in UBG). Collecting permits were issued by the corresponding regional governments: Junta de *Anda* *luciae* (UBG, SNS), Junta de Extremadura (JCB, HOR, COR, VER, SSP, DEL), Junta de Castilla y León (FRN, ADS, ADN, SAN, OMA, TUE) and Xunta de Galicia (LAS, LAR, ANC, MAC). All specimens were preserved in 100% ethanol for DNA extraction. Specimens were identified to species level using the taxonomic monographs for the European Chrysomelidae [19] and the Iberian Cryptocephalinae [18]. Male and female genitalia were dissected and mounted together with specimens using dimethyl hydantoin formaldehyde resin (DMHF). The careful inspection of genitalia was crucial for the robustness of morphological species diagnosis. Drawings were traced using CorelDraw X4 software, from images captured with a Nikon Coolpix 4500 digital camera attached to an Olympus SZX16 stereomicroscope.

**Table 1:** Collecting localities *of* *L* *. atricillus* or *L* *. bedelii* .

**Locality**	**Code**	**Latitude**	**Longitude**	**Number of specimens**	**Number of haplotypes**	**Species**	**Haplotype code**
Ancares	ANC	42.8257	-6.8811	1	1	*L* *. atricillus*	*atr_1877*
Valle del Tuéjar	TUE	42.8070	-4.9872	3	3	*L* *. atricillus*	*atr_34, atr_38, atr_1473*
Omaña	OMA	42.7869	-6.1411	3	1	*L* *. atricillus*	*atr_38*
Macizo Central	MAC	42.1869	-7.2053	1	1	*L* *. atricillus*	*atr_34*
Sanabria	SAN	42.0724	-6.6054	1	1	*L* *. atricillus*	*atr_1778*
Arribes del Duero-NORTE	ADN	41.5634	-6.1287	3	1	*L* *. atricillus*	*atr_38*
Arribes del Duero-SUR	ADS	41.0922	-6.7161	6	6	*L* *. atricillus*	*atr_34, atr_38, atr_57, atr_778, atr_1014, atr_1015*
La Vera	VER	40.0849	-5.7425	6	3	*L* *. atricillus*	*atr_34, atr_38, atr_778*
Deleitosa	DEL	39.6213	-5.5469	3	3	*L* *. atricillus*	*atr_38, atr_57, atr_256*
Sierra San Pedro	SSP	39.2014	-6.7741	2	2	*L* *. atricillus*	*atr_38, atr_ 1877*
PN Cornalvo	COR	39.0209	-6.1739	28	7	*L* *. atricillus*	*atr_34, atr_38, atr_56, atr_57, atr_93, atr_766, atr_767*
Sierra Norte de Sevilla	SNS	37.9413	-5.7113	3	2	*L* *. atricillus*	*atr_34, atr_38*
Ubrique-Grazalema	UBG	36.6148	-5.4239	2	2	*L* *. bedelii*	*bed_652, bed_714*

The geographic coordinates (latitude and longitude) are shown as well as the number of specimens and the number of haplotypes for each species. The code used to differentiate haplotypes is also provided.

**Table 2:** Collecting localities *of* *P* *. azureus* or *P* *. regius* .

**Locality**	**Code**	**Latitude**	**Longitude**	**Specimens# ( *P* *. azureus* )**	**Specimens# ( *P* *. regius* )**	**Haplotypes# ( *P* *. azureus* )**	**Haplotypes# ( *P* *. regius* )**	**Haplotype code**
Arribes del Duero-SUR	ADS	41.0922	-6.7161	4		4		*azu_1075, azu_1082, azu_1112, azu_1116*
Deleitosa	DEL	39.6213	-5.5469	7	2	6	2	*azu_246, azu_247, azu_253, azu_254, reg_284, azu_285, reg_296, azu_322*
Jerez de los Caballeros	JCB	38.3259	-6.7285	1	6	1	3	*azu_246, reg_879, reg_882, reg_904*
La Vera	VER	40.0849	-5.7425	11		10		*azu_453, azu_454, azu_456, azu_466, azu_467, azu_474, azu_500, azu_501, azu_515, azu_534*
PN Cornalvo	COR	39.0209	-6.1739	1	1	1	1	*azu_83, reg_84*
Sanabria	SAN	42.0724	-6.6054	1		1		*azu_1707*
Serra de Lastra	LAS	42.5146	-6.9292	8		8		*azu_83, azu_246, azu_254, azu_1234, azu_1243, azu_1258, azu_1259, azu_1290*
Sierra de Larouco	LAR	41.9995	-7.7062	6		6		*azu_246, azu_254, azu_1607, azu_1611, azu_1668, azu_1686*
Sierra de Francia	FRN	40.5046	-6.0609	7		6		*azu_246, azu_254, azu_375, azu_376, azu_404, azu_445*
Sierra de Hornachos	HOR	38.6045	-6.1046	1	9	1	3	*reg_882, reg_955, reg_957, azu_958*
Sierra San Pedro	SSP	39.2014	-6.7741	1	1	1	1	*azu_177, reg_214*
Valle del Tuéjar	TUE	42.8070	-4.9872	12		3		*azu_1453, azu_1494, azu_1545*

The geographic coordinates (latitude and longitude) are shown as well as the number of specimens and the number of haplotypes for each species. The code used to differentiate haplotypes is also provided.

### DNA sequencing and alignment

Genomic DNA was extracted from muscle tissue in the prothorax region with Wizard SV 96-well plates (Promega, UK). A 655 base pair region from the 5’ end of mitochondrial cytochrome oxidase I was amplified with primers CO1F2

(TCTACYAATCATAAAGATATTGGTAC) and CO1R2 (ACTTCTGGATGACCAAAGAATCA) in most cases or with standard Folmer LCO / HCO primers [22] when previous primers failed. Amplification conditions used with Bioline BioTaq were 95^°^C for 2 min, 35 cycles of 95^°^C for 30 s, 40^°^C for 30 s and 72^°^C for 45 s, and a final extension of 72^°^C for 5 min. Additionally, for some specimens a 823 bp fragment of the 3’ region of *cox1* was amplified with primers L2-N-3014 (Pat) and C1-J-2183 (Jerry) [23]; a 469 bp fragment of 16S rRNA with primers 16Sa and 16Sb; and a 1878 base pair fragment of the nuclear 18S rRNA gene with primers 18S5’, 18S5.0rw, 18Sai, 18Sbi, and 18sa2.0 and 18S3’I following Shull et al. [24]. Amplification conditions used with Bioline BioTaq were 94^°^C for 5 min, 35 cycles of 94^°^C for 45 s, 54^°^C (5’) or 52^°^C (3’) for 45 s and 72^°^C for 2 min, and a final extension of 72^°^C for 7 min. PCR products were cleaned with 96-well Millipore multiscreen plates and sequenced in both directions using ABI dye terminator sequencing. Sequence chromatograms were assembled and manually edited using Genious 5.6. Sequences are available under GenBank accession numbers KF134544 - KF134651.

### Phylogenetic analyses

To compare the intra- and inter-specific genetic variability in the studied species pairs with that of other congeneric species, we also included the most closely related species collected during the same sampling campaign (Table 3). Phylogenetic relationships were determined using Bayesian inference on a combined matrix from mitochondrial (*cox1-*3’, *cox1-*5’, 16S) and nuclear markers (18S). In the case of *Longitarsus*, we included 5 additional species that were recovered in the same clade as the studied pair, including *L* *. dorsalis* (Fabricius 1781), *L* *. ibericus* Leonardi & Mohr 1974, *L* *. luridus* (Scopoli 1763), *L* *. nigrocillus* (Motschulsky 1849) and *L* *. ochroleucus* (Marsham 1802), plus one specimen of *L* *. ordinatus* (Foudras 1860) as the outgroup. For *Pachybrachis*, we included in the analyses the two other collected species, *P* *. suffriani* Schaufuss 1862 and *P* *. terminalis* Suffrian 1849, plus one specimen of *Cryptocephalusoctoguttatus* (Linnaeus 1767) as the outgroup.

**Table 3:** Number of specimens and sampling localities for the close species included in the phylogenetic analyses.

	*L* *. dorsalis*	*L* *. ibericus*	*L* *. luridus*	*L* *. nigrocillus*	*L* *. ochroleucus*	*P* *. suffrianii*	*P* *. terminalis*
ANC		1	6				
ADN	1						
ADS							
DEL	17				2		
JCB	6						9
VER					3		
MAC			2			1	
OMA							
COR	16						
SAN						2	
LAS			11	1			
LAR							
FRN	1						
HOR				1			
SNS							
SSP	10				9		
UBG	11						
TUE			2				

The distributions of intra- and inter-specific p-distances were computed using the command *density* in R [25]. Maximum likelihood phylogenetic trees were built using unique haplotypes only. Gene trees were constructed with RAxML 7.0 [26] under the GTR+G+I model, which was selected by jModeltest [27]. The best tree and clade support values were computed using the rapid bootstrap algorithm with 100 replicates. To test the hypothesis of monophyly of the four focal species ( *L* *. atricillus* , *L* *. bedelii* , *P* *. azureus* , *P* *. regius* ) we performed Shimodaira-Hasegawa (SH) tests [28] using the R package *phangorn* [29]. The tests compared the log-likelihood of ML trees for *Longitarsus* and *Pachybrachis* species with those of constrained trees in which the topology was forced to preserve the monophyly of the focal species. Significant differences in log-likelihood values would imply that the support for non-monophyly is strong. Haplotype networks were created using TCS software [30] implemented in ANeCA v.1.2 [31]. TCS uses statistical parsimony to estimate haplotype networks of closely related individuals from DNA sequence data. The relationship between mtDNA genetic distance and geographical distance was assessed using Mantels tests [32]. We independently assessed the genetic-geographic distance relationships for pairs of conspecific specimens and pairs of interspecific specimens. In case of mitochondrial introgression, sympatric pairs of specimens should present more similar haplotypes than pairs collected at distant localities. This test was only conducted for *Pachybrachis*, but not for *Longitarsus* due to the low number of *L* *. bedelii* specimens.

## Results

A total of 143 individuals of the target species were collected (62 *L* *. atricillus* , 2 *L* *. bedelii* , 60 *P* *. azureus* , 19 *P* *. regius* ). DNA sequencing yielded a 655 bp fragment in all except for four specimens with shortened sequence (*reg_284*: 601 bp, *azu_474*: 643 bp; *reg_904*: 600 bp and *azu_1234*: 537 bp) and two specimens of *L* *. atricillus* for which PCR amplification failed completely. A total of 65 unique *cox1* haplotypes were detected (14 *L* *. atricillus* , 2 *L* *. bedelii* , 40 *P* *. azureus* , 9 *P* *. regius* ; see Tables 1 and 2). The two haplotypes of *L* *. bedelii* were only found at the southernmost locality. *Pachybrachis* *regius* was found at five localities in South-Central Spain. *Longitarsusatricillus* and *P* *. azureus* showed wide distributions across the Iberian transect. *Longitarsusatricillus* and *L* *. bedelii* were not collected together in the same localities despite our considerable sampling effort, while *P* *. azureus* and *P* *. regius* coexisted in five localities (Tables 1 and 2).

**Figure 2:**
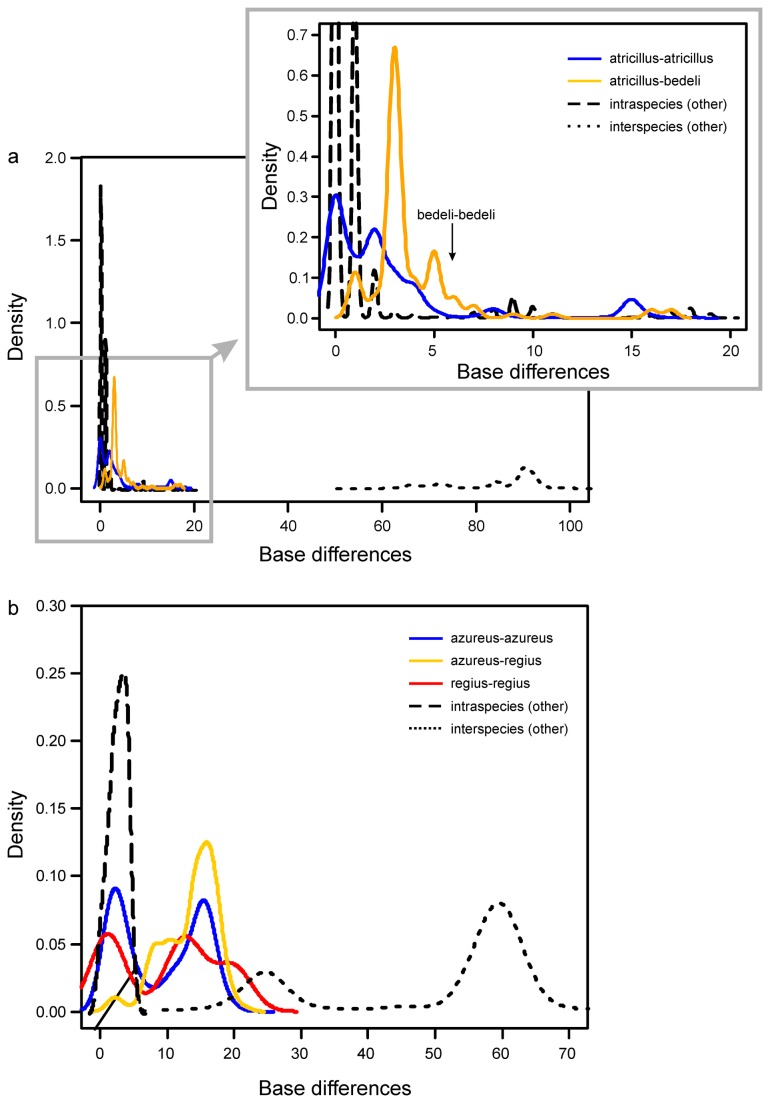
Density plots representing the distribution of divergence (number of different bases) between and within species for (a) *Longitarsus* and (b) *Pachybrachis*.

The *cox1* sequences of *L* *. atricillus* and *L* *. bedelii* showed variation in 33 nucleotide positions, most of which corresponded to differences within *L* *. atricillus* . Haplotypes of the two *L* *. bedelii* specimens were closely similar to some *L* *. atricillus* haplotypes: the minimum number of discrepancies between *L* *. bedelii* and *L* *. atricillus* was 1 base for *bed_652* and 3 bases for *bed_714*. The distributions of intraspecific and interspecific p-distances were broadly overlapping for *L* *. bedelii* and *L* *. atricillus* , a result not found in the remaining *Longitarsus* species (Figure 2a). The haplotype network analyses for *Longitarsus* species yielded six independent networks, one for each species except for *L* *. atricillus* and *L* *. bedelii* , which were included in a single network (Figure 1a). This network showed little geographic structure and showed no concordance with the morphological species, as both haplotypes belonging to *L* *. bedelii* appeared at opposite extremes in the network.

**Figure 3:**
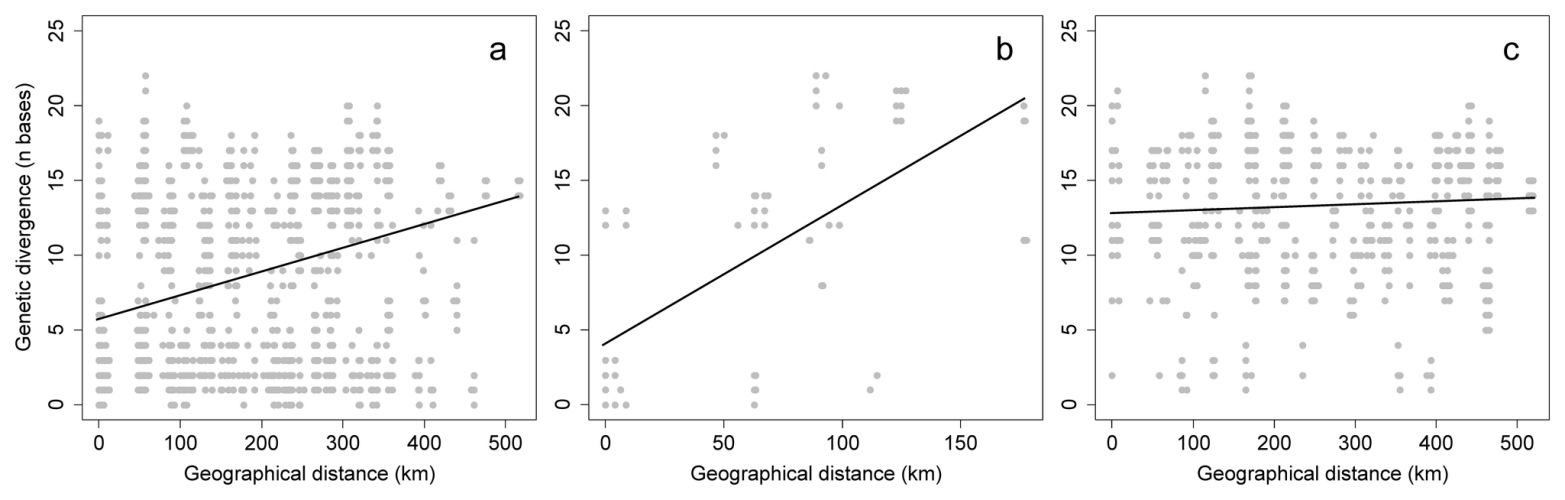
Relationship between genetic and geographic distance for (a) conspecific specimen pairs within *P* *. azureus* , (b) conspecific specimen pairs within *P* *. regius* , and (c) interspecific specimen pairs ( *P* *. azureus* */P. regius*). The straight lines are the fitted linear functions.

The alignment of *P* *. azureus* and *P* *. regius* *cox1* sequences revealed 77 variable positions. The distribution of intraspecific and interspecific variation was broadly overlapping for *P* *. azureus* and *P* *. regius* but not for the remaining species of *Pachbrachis* (Figure 2b). The minimum number of discrepancies between haplotypes of *P* *. azureus* and *P* *. regius* was 1 base. Again, the network analysis yielded an independent network for each species, except for *P* *. azureus* and *P* *. regius* that were included in a single network (Figure 1b). Little geographic structure was observed within this network, and specimens of both species appeared mixed in various portions of the network. Moreover, when the relationship between genetic and geographic distance was assessed (Figure 3), it turned out that although this relationship was weak but significant for conspecific specimens within *P* *. azureus* (r^2^=0.10, Mantel test p<0.001) and even more marked within *P* *. regius* (r^2^=0.40, Mantel test p<0.001), the relationship was negligible for interspecific specimen pairs (r^2^=0.0057, Mantel test p=0.005).

**Figure 4:**
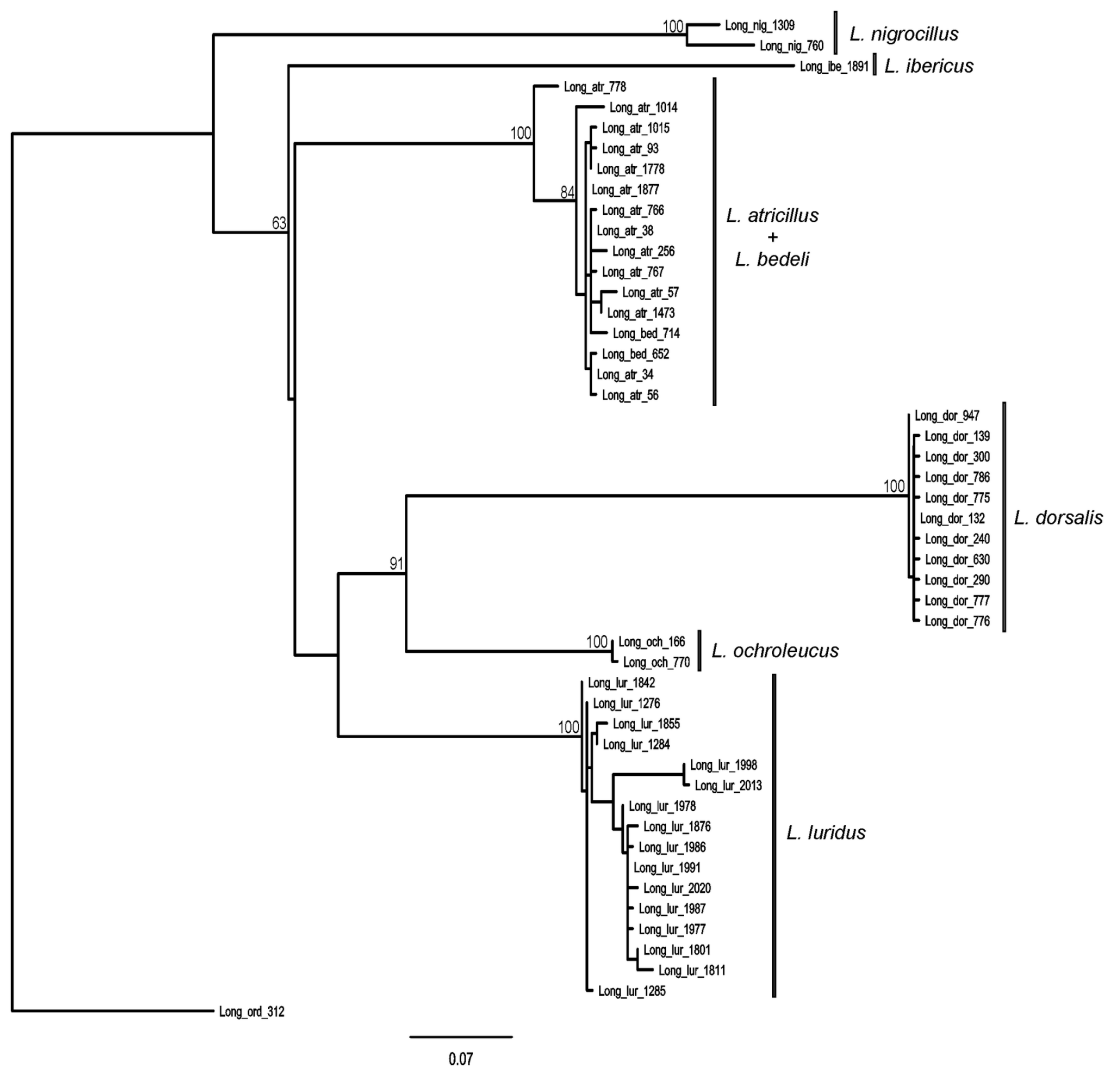
Maximum likelihood tree of *cox1*-5 ’ of *Longitarsus* spp. Node values are bootstrap support values.

**Figure 5:**
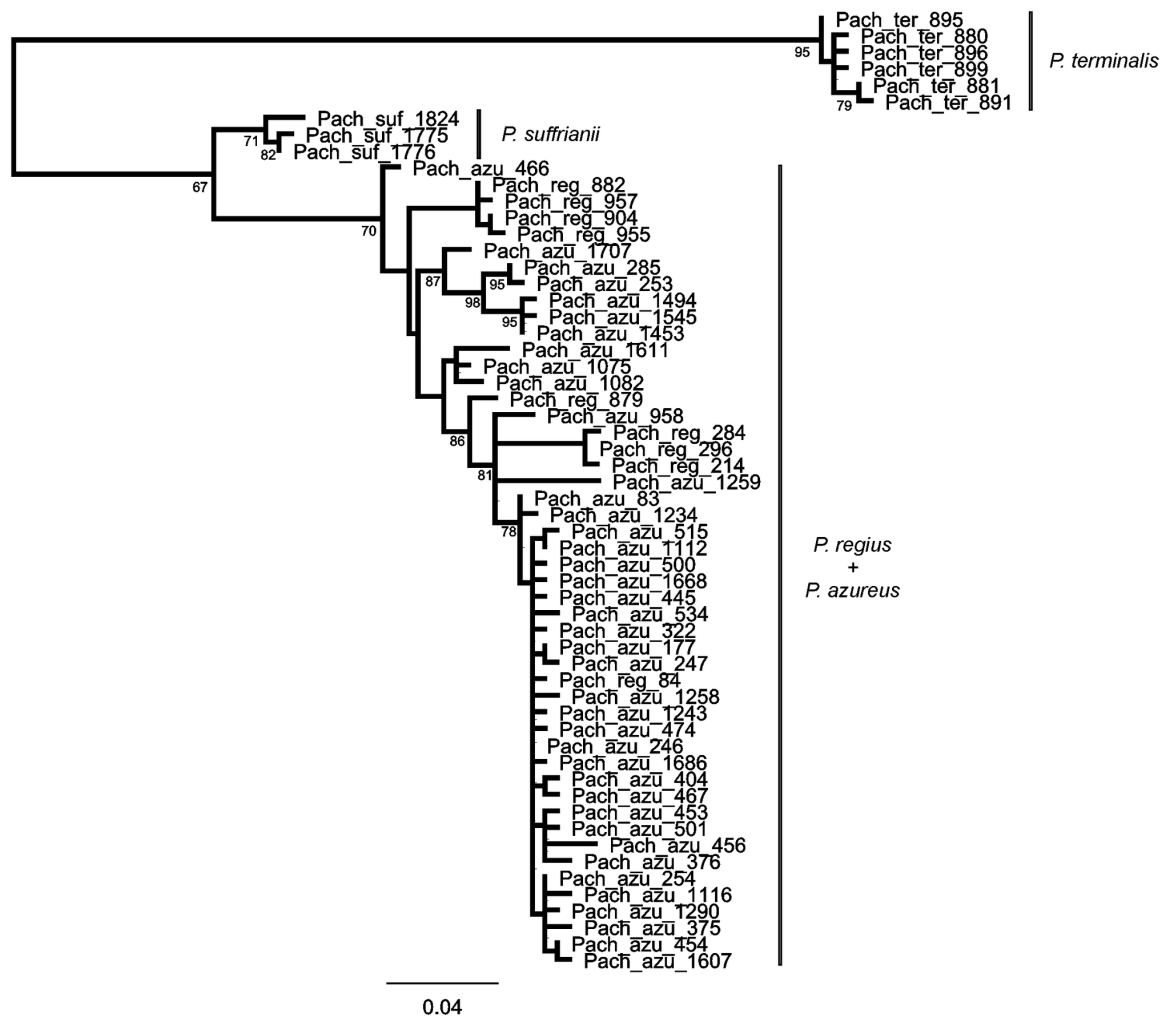
Maximum likelihood tree of *cox1*-5 ’ of *Pachybrachis* spp. Node values are bootstrap support values. That outgroup branch was removed to improve the visualization of the ingroup.

ML trees constructed from the *cox1* sequences did not show a clear separation between *L* *. atricillus* and *L* *. bedelii* (Figure 4) nor between *P* *. azureus* and *P* *. regius* (Figure 5). None of these species were recovered as monophyletic. This situation differed from all close congeneric species, which were recovered as monophyletic with high bootstrap support. The comparison of ML trees with those constrained for the reciprocal monophyly of the paired species revealed a significantly decreased log-likelihood (ML= -3316 versus -3340 in *Longitarsus*, and ML = -2612 to -2721 in *Pachybrachis*) in the SH tests (p<0.05 in both cases). This provided statistical support against the mononophyly of these species.

The available nuclear 18S sequences produced similar results. *L* *. atricillus* (8 samples) and *L* *. bedelii* (1 sample) were identical for the 18S fragment. In contrast, interspecific divergence between *L* *. atricillus* or *bedelii* and the other *Longitarsus* species ranged from 8 to 16 nucleotide changes. In *P* *. azureus* (7 samples) and *P* *. regius* (2 samples) 18S sequences differed at most in 2 bases (0 to 2 base changes within *P* *. azureus* and 0 to 1 divergence between *P* *. azureus* and *P* *. regius* ). This contrasted with the divergences between any specimens of these two species and those of *P* *. suffriani* (2 samples), which differed in 10 to 12 bases.

**Figure 6:**
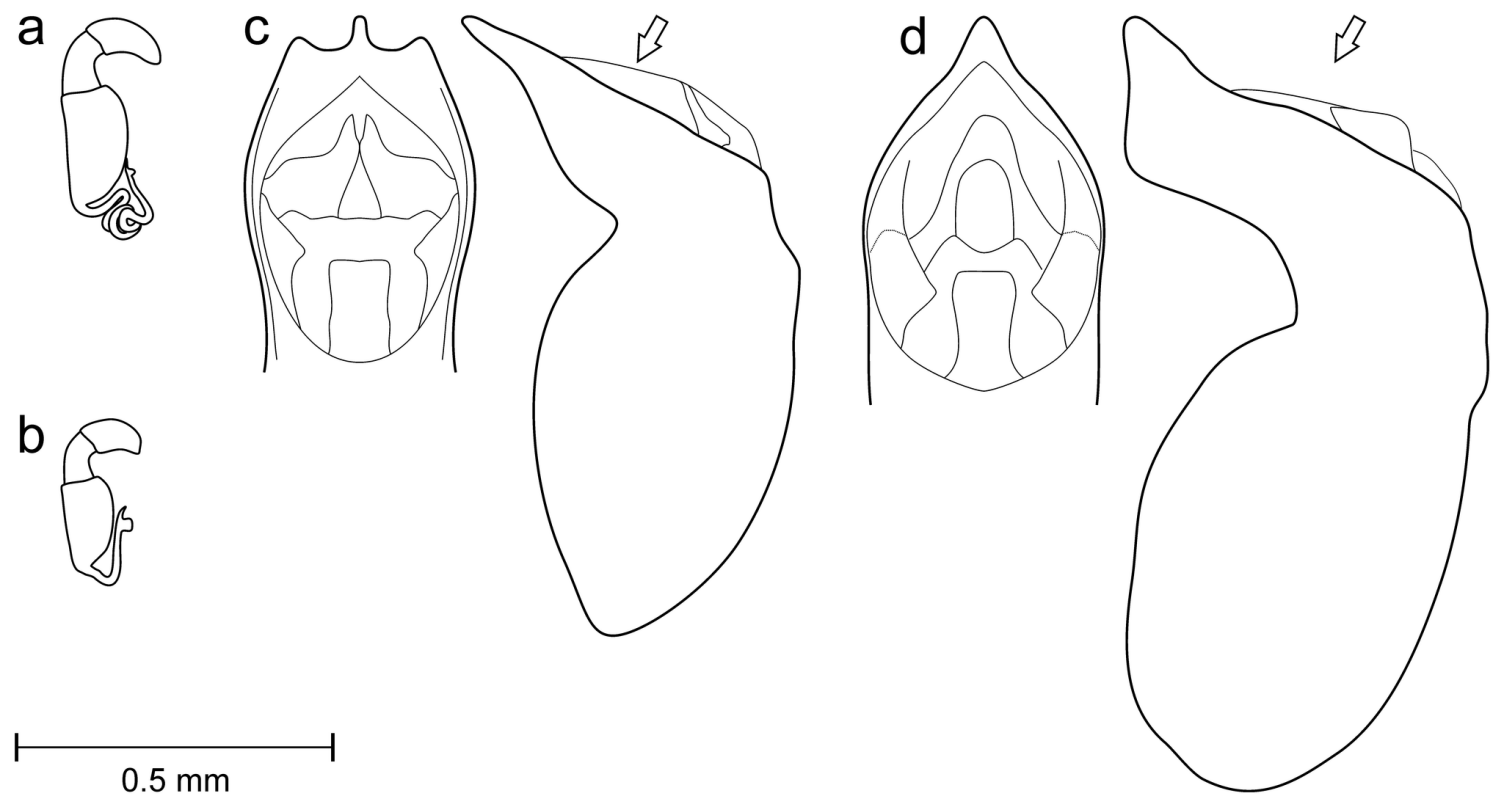
Morphological diagnostic characters. (a) Spermatheca of *L* *. atricillus* . (b) Spermatheca of *L* *. bedelii* . (c) Median lobe of aedeagus of *P* *. azureus* . (d) Median lobe of aedeagus of *P* *. regius* .

Both of the *Longitarsus* and *Pachybrachis* species in the analysed pairs showed clear diagnostic morphological characters. The *Longitarsus* specimens were unequivocally attributed either to *L* *. atricillus* or *L* *. bedelii* based on the elytral coloration (pale yellowish brown in the former versus dark brown to black with an apical yellowish spot in the latter) and especially based on female genitalia (spermathecal duct with numerous loops versus spermathecal duct simply arched; Figure 6a-b). The *Pachybrachis* specimens strictly conformed to the well known descriptions of either *P* *. azureus* or *P* *. regius* that can be easily separated based on the colour of the last elevated elytral interstria (metallic in the former versus yellow, at least in part, in the latter) and especially by the shape of the median lobe of aedeagus (apically tridentate in the former versus acuminated in the latter; Figure 6c-d). No intermediate character states were observed.

## Discussion

Our results revealed two pairs of Iberian leaf beetles, *L* *. atricillus* / *L* *. bedelii* and *P* *. azureus* / *P* *. regius* that exhibit consistent morphological differences pointing to the existence of well defined species. In contrast, within each congeneric pair, interspecific divergences in mtDNA were within the range of intraspecific divergences of 2.8% and 3.4%, respectively, but revealed no discernible phylogenetic structure that would separate the species within each pair. In addition, we did not observe any obvious pattern linking the haplotype divergence to geographic distance or known landscape barriers. The high intraspecific divergence suggests great lineage age, while the lack of geographical structure and the existence of a few widespread haplotypes points to some level of dispersal among the studied populations. In both cases, the nuclear 18S rRNA also did not split these lineages any further, but other congeneric species were clearly distinguishable.

In the two species of *Longitarsus*, major morphological differences include elytral coloration and markedly dissimilar female genitalia. No morphological intermediates have been described anywhere in the range of either type. This corroborates the existence of two evolutionarily independent species, and not simply intraspecific variability. The known spatial ranges of both species are widely overlapping in the western half of the Iberian Peninsula. *Longitarsusatricillus* has been recorded in almost all regions of the Iberian Peninsula [33,34], while *L* *. bedelii* is an Iberian endemic described from Dehesa de Malpica de España (38.77^°^ N, 7.15^°^ W) in the surroundings of Badajoz, southwestern Spain [35] and more recently was recorded from several localities in western Spain, including Asturias (the type locality of the *L* *. danieli* Mohr 1962, a junior synonym of *L* *. bedelii* after Bastazo [36]), Ourense, Zamora, León and Ávila [33,37–41]. No spatial or environmental factors are likely to be associated with the different morphologies, although host plant information is lacking for *L* *. bedelii* . These observations are in accordance with the haplotype network that also implies the absence of any geographical structure associated to particular morphologies.

The evidence for two independent evolutionary lineages is even clearer in the case of *P* *. azureus* and *P* *. regius* . The distributions of both species overlap throughout the range of *P* *. regius* [18], and we collected specimens attributed to both species in strict sympatry. The morphological differences between both species are very clearly marked. The genital diagnostic characters, as well as other external characters such as elytral coloration, are known to remain constant across the ranges of both species and no hybrid specimens have been documented. Therefore, the existence of discrete diagnostic characters in both congeneric pairs supports the notion that no genetic exchange occurs between the morphological forms, as suggested by the molecular data.

The discordance of morphology and mtDNA would require further investigation of nuclear markers to test for the existence of differential gene flow or selective extinction limited to mtDNA. The available sequences for the nuclear 18S rRNA gene showed little variation within each pair. All other congeneric species did show interspecific divergences for this nuclear marker, which demonstrates that 18S rRNA is generally useful for species discrimination despite its slow rate of variation. Yet, when species are very closely related, the power of this marker becomes limited and therefore it is not clear if the lack of differentiation within these two species pairs is simply due to the slow rate, which renders the marker uninformative.

Most cases of mitochondrial introgression are evident from the presence of ‘foreign’ haplotypes within the range of a species, which affects only a small portion of the total range [11]. In contrast, incomplete lineage sorting is not expected to result in any predictable distribution within the range of the ancestral species [11]. We showed here a complete lack of genetic differentiation with geographic distance in each of the two species pairs (Figure 3), which supports a scenario of incomplete lineage sorting. The high intraspecific variation in each of the species pairs also attests to a long history since the joint origin of both species, suggesting a single, large gene pool relating to both species in the pair, from which the morphological types were drawn fairly recently, while processes of lineage sorting for either group are lagging behind the clear morphological and presumably reproductive separation. Because the mtDNA haplotypes in the species with narrower ranges in both pairs are unique, some level of variation may have accumulated in these groups since they originated from the ancestral gene pool. While more nuclear genes need to be analyzed to confirm the patterns, the simplest explanation is that stochastic lineage sorting resulted in the distribution of particular haplotype lineages in either of the morphological species. If populations are rather stable, as can be expected in the Iberian Peninsula [42], lineage sorting of mtDNA due to drift may be slow, while processes of species formation due to divergent natural or sexual selection may continue at a rapid pace, leading to the origin of morphologically distinct taxa.

In practical terms, based on the DNA data, these morphologically separate species are neither monophyletic nor diagnosable (even allowing for a paraphyletic species concept [43]), and therefore haplotype similarity within species is not greater than between species. Hence there is no “barcoding gap” for easy recognition of differentiated species. This finding might suggest that mtDNA-based community surveys are compromised by morphological-molecular incongruence, but for Iberian leaf beetle communities these cases are a small minority in the sample of >200 species and would not distort the large-scale patterns (Baselga et al., unpublished). The fact that only a few species in this survey produced this kind of morphological-molecular incongruence makes the discovery of these cases noteworthy. They differ from commonly encountered situations because mitochondrial incongruence does not appear to be limited to partial ranges. In particular, our results are interesting because variation in both mitochondrial and nuclear markers was discordant with morphological differentiation. As male and female genital characters differed markedly in these species pairs, this system may be a promising opportunity to pursue an understanding of rapid evolution of secondary sexual characteristics driven by sexual selection.
