# Correction: Rare Failures of DNA Bar Codes to Separate Morphologically Distinct Species in a Biodiversity Survey of Iberian Leaf Beetles

**DOI:** 10.1371/annotation/18a13c3f-ea86-45bf-9934-35d9bde2029c

**Published:** 2013-09-18

**Authors:** Andrés Baselga, Carola Gómez-Rodríguez, Francisco Novoa, Alfried P. Vogler

As a result of an error in the typesetting process, there was an error in the title of the article.

The correct title is: Rare Failures of DNA Barcodes to Separate Morphologically Distinct Species in a Biodiversity Survey of Iberian Leaf Beetles

The correct citation is: Baselga A, Gómez-Rodríguez C, Novoa F, Vogler AP (2013) Rare Failures of DNA Barcodes to Separate Morphologically Distinct Species in a Biodiversity Survey of Iberian Leaf Beetles. PLoS ONE 8(9): e74854. doi:10.1371/journal.pone.0074854 

